# The natural organosulfur compound dipropyltetrasulfide prevents HOCl-induced systemic sclerosis in the mouse

**DOI:** 10.1186/ar4351

**Published:** 2013-10-28

**Authors:** Wioleta Marut, Vincent Jamier, Niloufar Kavian, Amélie Servettaz, Paul G Winyard, Paul Eggleton, Awais Anwar, Carole Nicco, Claus Jacob, Christiane Chéreau, Bernard Weill, Frédéric Batteux

**Affiliations:** 1 Université Paris Descartes, Faculté de Médecine, EA 1833 et Laboratoire d’Immunologie Biologique, Hôpital Cochin AP-HP, 75679 Paris, cedex 14, France; 2 Division of Bioorganic Chemistry, School of Pharmacy, Saarland University, Campus B2.1, D-66123 Saarbrücken, Germany; 3 Faculté de Médecine de Reims, Service de Médecine Interne, Maladies Infectieuses, Immunologie Clinique, Hôpital Robert Debré, 51092 Reims, cedex, France; 4 Exeter University Medical School, University of Exeter, EX1 2LU Exeter, Devon, UK; 5 ECOspray Limited, Grange Farm Hilborough, Thetford IP26 5BT UK

## Abstract

**Introduction:**

The aim of this study was to test the naturally occurring organosulfur compound dipropyltetrasulfide (DPTTS), found in plants, which has antibiotic and anticancer properties, as a treatment for HOCl-induced systemic sclerosis in the mouse.

**Methods:**

The prooxidative, antiproliferative, and cytotoxic effects of DPTTS were evaluated *ex vivo* on fibroblasts from normal and HOCl mice. *In vivo,* the antifibrotic and immunomodulating properties of DPTTS were evaluated in the skin and lungs of HOCl mice.

**Results:**

H_2_O_2_ production was higher in fibroblasts derived from HOCl mice than in normal fibroblasts (*P* < 0.05). DPTTS did not increase H_2_O_2_ production in normal fibroblasts, but DPTTS dose-dependently increased H_2_O_2_ production in HOCl fibroblasts (*P* < 0.001 with 40 μ*M* DPTTS). Because H_2_O_2_ reached a lethal threshold in cells from HOCl mice, the antiproliferative, cytotoxic, and proapoptotic effects of DPTTS were significantly higher in HOCl fibroblasts than for normal fibroblasts. *In vivo,* DPTTS decreased dermal thickness (*P* < 0.001), collagen content in skin (*P* < 0.01) and lungs (*P* < 0.05), αSMA (*P* < 0.01) and pSMAD2/3 (*P* < 0.01) expression in skin, formation of advanced oxidation protein products and anti-DNA topoisomerase-1 antibodies in serum (*P* < 0.05) versus untreated HOCl mice. Moreover, in HOCl mice, DPTTS reduced splenic B-cell counts (*P* < 0.01), the proliferative rates of B-splenocytes stimulated by lipopolysaccharide (*P* < 0.05), and T-splenocytes stimulated by anti-CD3/CD28 mAb (*P* < 0.001). *Ex vivo,* it also reduced the production of IL-4 and IL-13 by activated T cells (*P* < 0.05 in both cases).

**Conclusions:**

The natural organosulfur compound DPTTS prevents skin and lung fibrosis in the mouse through the selective killing of diseased fibroblasts and its immunomodulating properties. DPTTS may be a potential treatment for systemic sclerosis.

## Introduction

Systemic sclerosis is a connective tissue disease characterized by fibrosis of skin and visceral organs, vascular disorders, and dysimmunity [1]. Although the pathogenesis of systemic sclerosis is not fully understood, recent data suggested that oxidative stress and inflammation play an important role in the initiation and development of this disease [2-4]. At an early stage of systemic sclerosis, activated fibroblasts constitutively produce high amounts of reactive oxygen species (ROS) that cause the synthesis of type I collagen and lead to fibrosis [3]. The release of highly toxic ROS by activated fibroblasts and endothelial cells induces an inflammatory process that triggers the recruitment of inflammatory cells, the production of cytokines, and increases the fibrotic process [5] through the involvement of the RAS/MAP kinase pathways [6]. In our mouse model of systemic sclerosis (induced by HOCl), an activated phenotype, an overproduction of ROS, and a drop in the content of reduced glutathione are observed in diseased fibroblasts (3, 14). The involvement of the immune system in the pathogenesis of SSc is also reflected by circulating auto-antibodies, such as anti-DNA topoisomerase-1 antibodies (Abs) that are characteristic of diffuse SSc and consecutive to a breach of tolerance caused by oxidized DNA topoisomerase-1 [7]. Auto-abs against platelet-derived growth factor receptor are also found in SSc, that trigger the production of ROS and can play a role in the perpetuation of the disease. If intracellular ROS can stimulate cell growth and fibrosis, ROS can also lead to cell death beyond a certain level of intracellular production. ROS generating molecules such as arsenic trioxide can kill fibroblasts in constitutively activated SSc, thus abrogating the development of fibrosis in two mouse models of SSc. However, the compounds used so far have generated several side effects that have limited their use in SSc. Dipropyltetrasulfide (DPTTS) is a natural organosulfur compound found in *Allium,* that is endowed with pro-oxidative properties and is considered as an antibiotic or anti-mitotic agent independently of its effects on oxidative stress [8,9]. Polysulfides such as DPTTS, are already considered as a promising new class of antibiotics for resistant bacteria [10]. In this study, we investigated the effects of DPTTS on skin fibrosis and immune dysregulations in HOCl-induced SSc in the mouse.

## Methods

### Animals, chemicals, and procedure

Six-week-old female BALB/c mice were used in all experiments (Harlan, Gannat, France). All mice received humane care according to our institutional guidelines. Mice underwent an intradermal injection of 300 μl of a solution generating HOCl into their back every day for 6 weeks. The same number of mice received PBS under the same conditions and times as controls. One week after injection, the animals were killed by cervical dislocation. Serum and tissue samples were collected from each mouse and stored at -80°C until use. This study was conducted in compliance with approved animal experimental procedure number 11-32/11-33, accorded by the French *Comité d'Ethique en Matière d'Expérimentation Animale Paris Descartes (CEEA 34).*

HOCl was produced by adding 166 μl of NaClO solution (9.6% as active chlorine) to 11.1 ml of KH_2_PO_4_ solution (100 m*M* (pH 6.2)) (16). The HOCl concentration was determined by spectrophotometry at 280 nm (molar absorption coefficient = 350 μM^/^/cm) The optical density (OD) at 280 nm was adjusted to 0.7 to 0.9, and the amount of sodium hypochlorite and/or KH_2_PO_4_ solution was adjusted to retain the optimal HOCl concentration generated, based on the OD. All cells were cultured as reported previously [7]. All chemicals were from Sigma-Aldrich (France), if not specified.

### Synthesis of dipropyltetrasulfide

Dipropyltetrasulfide (DPTTS) was synthesized from propylmercaptan and sulfur chloride (S_2_Cl_2_). A solution of 10 m*M* propylmercaptan and 10 m*M* pyridine in 25 ml anhydrous diethyl ether was stirred at -78°C. A solution of 10 mM sulfur monochloride in 50 ml anhydrous diethyl ether was added dropwise over a period of 0.5 hours. The reaction mixture was stirred for an additional 0.5 hours, and another solution of 10 m*M* propylmercaptan and 10 m*M* pyridine in 25 ml anhydrous diethyl ether was added dropwise over a 0.5–hour period. The reaction mixture was stirred for an additional hour. The reaction was stopped by adding 25 ml of H_2_O. The mixture was brought to room temperature, and then adjusted with 0.5 *M* NaOH until the pH was neutral, pH 7. The organic phase was dried over MgSO_4_, filtered, and evaporated to yield a yellow oil with a strong onion smell. DPTTS was purified with column chromatography by using petrol ether:chloroform (95:3) as eluent. Characterization of the compound was carried out by NMR (Bruker Rheinstetten) type DRX 500 and Avance 500); ^1^H NMR (500 MHz, CDCl3): δ1.02 (6H, t, *J =* 7.4 Hz), 1.79 (m, 4H), 2.91(4H, t, *J* = 7.4 Hz). The molecular mass was confirmed by GC-MS, and purity was confirmed with HPLC. The MS values obtained were m/z 214 (M+), 184, 150, and 75 [11].

### Isolation of fibroblasts from the skin of mice

At the time of death, skin fragments were collected from HOCl-treated mice or PBS-treated mice. The fragments of skin were digested with “liver digest medium” (Invitrogen) for 1 hour at 37°C. After three washes, isolated cells were seeded into sterile flasks, and isolated fibroblasts were cultured in DMEM/Glutamax-I supplemented with 10% heat-inactivated fetal calf serum and antibiotics at 37°C in humidified atmosphere with 5% CO_2_, as previously described [7].

### H_2_O_2_ production and levels of intracellular reduced glutathione

The 4 × 10^4^ cells/well of isolated normal and HOCl^-^ fibroblasts were coated in 96-well plates (Costar) and incubated for 48 hours at 37°C with either medium alone or with 2.5, 5, 10, 20, or 40 μ*M* DPTTS. Levels of H_2_O_2_ and GSH were assessed spectrofluorometrically (Fusion; Perkin Elmer, Wellesley, MA, USA) by using 2′, 7′-dichlorodihydrofluorescein diacetate (H_2_DCFDA) and monochlorobimane, respectively. Here, cells were incubated with 200 μ*M* H_2_DCFDA for 1 hour or 50 μ*M* monochlorobimane in PBS for 15 minutes at 37°C. Intracellular H_2_O_2_ and GSH levels were expressed as arbitrary units of fluorescence intensity referred to the number of viable cells as assessed with the Crystal Violet assay.

### Modulation of H_2_O_2_ metabolism in normal and SSc fibroblasts

Isolated primary fibroblasts (2 × 10^4^ cells/well) from normal and HOCl mice were seeded in 96-well plates and incubated for 12 hours in complete medium alone or with the following molecules: 3.2 m*M N*-acetylcysteine (NAC, a GSH precursor), 1.6 m*M* BSO (GSH inhibitor), 20 U PEG-catalase, 400 μ*M* aminotriazol, catalase inhibitor), or 8 μ*M* diethyldithiocarbamate (DDC, superoxide dismutase inhibitor). DPTTS (30 μ*M*) was added during the last 16 hours. Cells were then washed 3 times with PBS and incubated with 100 μl per well of 200 μ*M* H_2_DCFDA for 30 minutes. Intracellular H_2_O_2_ levels were expressed as described earlier.

### *In vitro* cell-proliferation and viability assays

Isolated normal and HOCl fibroblasts (4 × 10^3^ cells/well) (Costar) were incubated in 96-well plates with complete medium and various doses of DPTTS (10 to 40 μ*M*) for 48 hours at 37°C. Cell proliferation was determined by pulsing the cells with [^3^H]thymidine (1 μCi/well) during the last 16 hours of culture, as previously described [7]. Cell viability was evaluated with the CV assay [12]. Results are expressed as percentages of viable treated cells *versus* viable untreated cells.

### Fluorescence-activated cell-sorting analysis of cell death

Apoptosis and necrosis were analyzed with the fluorescence-activated cell-sorting (FACS) Canto II flow cytometer (Becton Dickinson), by using the Membrane Permeability/Dead Cell Apoptosis Kit with YO-PRO-1 and propidium iodide (PI) for flow cytometry (Invitrogen), according to the manufacturer’s recommendations. In brief, isolated normal and HOCl fibroblasts (1.2 × 10^4^) were incubated with 40 μ*M* DPTTS for 5, 10, 15, or 24 hours. After the incubation period, cells were collected, washed 2 times with PBS, stained for 10 minutes on ice with 1.5 μ*M* PI and 0.1 μ*M* YO-PRO-1, and analyzed with flow cytometry.

### Dermal thickness

Skin thickness was measured on the backs of the mice in the area of intradermal injections 1 day before killing. Dermal thickness was measured with a caliper and expressed in millimeters [7].

### Measurements of collagen content in skin and lung

Skin was taken with a punch (6 mm diameter), and lung pieces were diced using a sharp scalpel, mixed with pepsin (1:10 weight ratio) and 0.5 *M* acetic acid at room temperature. After 3 days, collagen content was assayed by using the quantitative dye-binding Sircol method (Biocolor, Belfast, N. Ireland) [13,14].

### *Ex vivo* skin fibroblast proliferation

Primary normal and HOCl fibroblasts from HOCl mice or PBS mice treated or not with DPTTS (4 × 10^3^ cells/well) (Costar; Corning, Inc., Corning, NY, USA) were incubated in 96-well plates with complete medium, for 48 hours at 37°C. Cell proliferation was determined by pulsing the cells with [^3^H]thymidine (1 μCi/well) during the last 16 hours of culture, as described earlier.

### Histopathologic analysis

A 5-μm-thick tissue section was prepared from the mid-portion of paraffin-embedded skin and lung pieces and stained with hematoxylin/eosin. Slides were examined with standard bright-field microscopy (Olympus BX60) by a pathologist who was blinded to the assignment of the animal.

### Analysis of α-SMA and pSmad2/3 expression in mouse skin

Expression of α-SMA and pSmad2/3 was analyzed with immunohistochemistry of skin fragments derived from HOCl and PBS mice treated or not with DPTTS. Tissue sections were deparaffinized and rehydrated, and then incubated with 200 μg/ml proteinase K for 15 minutes at 37°C for antigen retrieval. Specimens were then treated with 3% vol/vol H_2_O_2_ for 10 minutes at 37°C to inhibit endogenous peroxidases and then blocked with BSA 5% wt/vol for 1 hour at 4°C. Sections were incubated with 1:100 anti-α-smooth muscle actin, mAb conjugated with alkaline phosphatase (Sigma-Aldrich) and with a 1:100 mAb directed to phospho-Smad2/3 (Cell Signaling Technology) for 2 hours at room temperature. Sections incubated with pSmad2/3 were then incubated with HRP-conjugated secondary goat anti-rabbit ab (Rockland) for 1 hour at room temperature. Antibody binding for αSMA staining was visualised by using nitroblue tetrazolium chloride/5-bromo-4-chloro-3-indolyl phosphate (NBT/BCIP). Staining of pSmad2/3 was visualized by using diaminobenzidine tetrahydrochloride (DAB) as a chromogen. The slides were examined with standard bright-field microscopy (Olympus BX60). Appropriate controls with irrelevant alkaline phosphatase-conjugated and HRP-conjugated abs were performed.

### Determination of advanced oxidation protein product (AOPP) concentrations in sera

AOPP were measured with spectrophotometry, as previously described [7]. Calibration used chloramine-T within the range of 0 to 100 Μ.

### Detection of serum anti-DNA topoisomerase-1 IgG Abs

Serum levels of anti-DNA topoisomerase-1 IgG abs were detected with ELISA by using coated DNA topoisomerase-1 purified from calf thymus (Immunovision). Optical density was measured at 405 nm by using a Dynatech MR 5000 microplate reader (Dynex Technology).

### Flow-cytometric analysis and splenocyte proliferation

Spleen cell suspensions were prepared after hypotonic lysis of erythrocytes. Splenocytes (10^6^ cells) were incubated with 1:200 anti-B220-PE antibody for 30 minutes at 4°C. Cells were then analyzed with a FACS Canto flow cytometer (BD Biosciences). For spleen cell proliferation, B and T cells were purified with MACS and were coated onto 96-well plates. In brief, splenic B- or T-cell suspensions (2 × 10^5^ cells) were cultured with 10 μg/ml of LPS (Boehringer, Mannheim, Germany) for B cells, or with 2.5-μg/ml precoated anti-CD3 and 1-μg/ml precoated anti-CD28 mAbs for T cells. Cell proliferation was determined as described earlier.

### Determination of IL-4 and IL-13

MACS-purified splenic T cells (2 × 10^5^ cells per well) were cultured in 96-well plates in complete medium for 48 hours at room temperature in the presence of 5 μg/ml concanavalin A. Cytokines were measured from the collected supernatants with ELISA (R&D Systems) by following the manufacturer’s instructions. Determined concentrations were expressed in nanograms per milliliter.

### Statistical analysis

All quantitative data were expressed as mean ± SEM. Data were compared by using one-way ANOVA plus the Tukey test for the comparison of means among multiple groups. *P* value of <0.05 was considered significant.

## Results

### DPTTS exerted *in vitro* antiproliferative and cytotoxic effects on normal and HOCl fibroblasts

**Figure 1:**
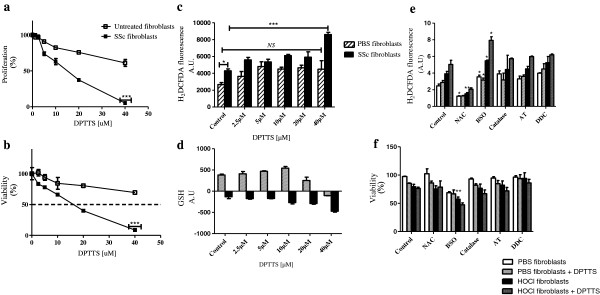
**Effect of DPTTS on normal fibroblasts and fibroblasts from HOCl mice *in vitro*. (a)** Proliferation of normal and HOCl fibroblasts incubated with DPTTS. Cellular proliferation was measured with thymidine incorporation. Results are expressed as percentages of viable treated cells *versus* untreated cells. **(b)** Cytotoxic effects of DPTTS. The viability of fibroblasts was determined with Crystal Violet assay. **(c)** Production of ROS (H_2_O_2_) as analyzed spectrofluorimetrically by using H_2_DCFDA. **(d)** Intracellular glutathione levels as analyzed spectrofluorimetrically by using monochlorobimane. **(e, f)** Primary normal and HOCl fibroblasts (three mice per group) were seeded in a 96-well plate in triplicates (2 × 10^4^/well) with various modulators (NAC, BSO, catalase, aminotriazole, DDC) for 12 hours and exposed to DPTTS for 16 hours. Production of ROS (H_2_O_2_) **(e)** and viability (F) were determined as expressed earlier. Results are given as the mean ± SEM of three independent experiments. *P < 0.05; **P < 0.01; ***P < 0.001.

Fibroblasts from normal and HOCl mice were exposed *in vitro* to increasing amounts of DPTTS (10 μ*M* to 40 μ*M*). The proliferative rates of HOCl fibroblasts were 62.9% ± 4.2% and 5.1% ± 0.5% in the presence of 10 μ*M* and 40 μ*M* DPTTS, respectively. These rates were lower than those found for PBS-fibroblasts under the same conditions (82.4 ± 1.4%, *P* < 0.01 and 61.1 ±4.8, *P* < 0.001). Thus DPTTS exerted a stronger antiproliferative effect on HOCl fibroblasts than on normal fibroblasts (Figure 1a). Similarly the cytotoxic effects of DPTTS were higher against HOCl fibroblasts than against normal fibroblasts, because the viability rates of HOCl fibroblasts were 66.1% ± 2.2% and 8.6% ± 4.7% in the presence of 10 μ*M* and 40 μ*M* DPTTS, respectively, versus 84.3% ± 9.5%; *P* < 0.05; and 69.4% ± 1.1% (*P* < 0.001) under the same conditions (Figure 1b).

### DPTTS exerted prooxidative effects *in vitro*

The basal production of H_2_O_2_ was increased by 39% in HOCl fibroblasts compared with normal fibroblasts (4,285 A.U. ± 241 versus 2,658 A.U. ± 235; *P* < 0.05; Figure 1c). Incubation of normal fibroblasts with DPTTS did not increase significantly the production of H_2_O_2_. In contrast, DPTTS dose-dependently increased the production of H_2_O_2_ by HOCl fibroblasts (8,595 A.U. ± 269 versus 4,285 A.U. ± 241; *P* < 0.001, with 40 μ*M* DPTTS; Figure 1c). We also investigated the effects of DPTTS on the level of reduced glutathione (GSH), an essential substrate involved in H_2_O_2_ catabolism. The basal level of reduced GSH was decreased by 166% ± in HOCl fibroblasts compared with normal fibroblasts (-132 A.U. ± 43.8 versus 380 A.U. ± 22.6; *P* < 0.001, Figure 1d). The level of intracellular glutathione was significantly higher (*P* < 0.001) in normal fibroblasts than in HOCl fibroblasts in the presence of DPTTS at all tested doses (Figure 1d).

### Modulation of H_2_O_2_ metabolism in SSc fibroblasts

We next investigated the mechanism of action of DPTTS by using specific modulators of oxidative stress. PBS or HOCl fibroblasts were incubated with or without DPTTS in the presence of NAC, BSO, catalase, AT, or DDC. Coincubation of DPTTS with NAC, a precursor of GSH, significantly decreased H_2_O_2_ production by 57% in PBS fibroblasts and by 60% in HOCl fibroblasts (*P <* 0.05 for normal and *P <* 0.01 for HOCl fibroblasts, Figure 1e). Hydrogen peroxide is converted into H_2_O by catalase and the GSH/GPx complex. Depleting GSH with BSO significantly increased H_2_O_2_ production by 30% in HOCl fibroblasts (*P* < 0.05) and by 31% in PBS fibroblasts (*P* < 0.05). In addition, H_2_O_2_ production by HOCl fibroblasts coincubated with DPTTS and BSO reached 7.92 ± 0.4 A.U. compared with those incubated with BSO alone (5.48 ± 0.08 A.U.) or DPTTS alone (3.15 ± 0.3 A.U.), showing the additive effect of DPTTS and BSO (*P <* 0.05). Conversely, addition of DPTTS in the presence of the catalase inhibitor ATZ or with exogenous PEG-catalase or with the superoxide dismutase inhibitor DDC had no effect on the levels of H_2_O_2_ in normal and HOCl fibroblasts (Figure 1e). Depleting GSH by adding BSO to the culture medium with DPTTS significantly decreased the viability of HOCl fibroblasts (*P* < 0.01). In contrast, specific inhibition of catalase by ATZ or of superoxide dismutase by DDC had no effect on the viability of normal and HOCl fibroblasts (Figure 1f).

### DPTTS-induced apoptosis in PBS and HOCl fibroblasts

**Figure 2:**
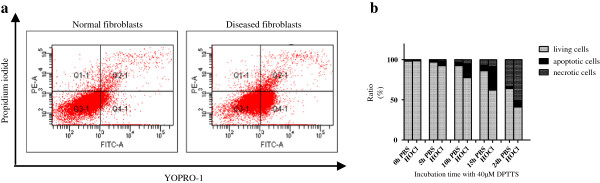
**Proapoptotic effects of DPTTS analyzed with fluorescence-activated cells sorting. (a)** Normal and HOCl fibroblasts were incubated with 40 μ*M* DPTTS for 5, 10, or 15 hours. The ratio of apoptosis to necrosis was analyzed with flow cytometry by using the Membrane Permeability/Dead Cell Apoptosis Kit with YO-PRO-1 and propidium iodide. Necrotic cells were PI positive, and apoptotic cells were YO-PRO-1 positive. One of three representative experiments is shown. **(b)** Kinetics of the apoptosis/necrosis ratio of normal and HOCl fibroblasts treated with 40 μ*M* DPTTS.

Fibroblasts extracted from the skin of PBS and of HOCl mice were incubated with 10, 20, and 40 μ*M* DPTTS for 5, 10, 15, or 24 hours. The highest level of cytotoxicity was observed with 40 μ*M* DPTTS. After 15 hours at this concentration, the viability was decreased by 38% in HOCl fibroblasts and by 14% in PBS fibroblasts (*P* < 0.001; Figure 2a). A kinetic analysis of cell death between 5 and 24 hours showed that DPTTS mediated cell death essentially through an apoptotic process (Figure 2b).

### DPTTS decreased skin and lung fibrosis in mice with SSc

**Figure 3:**
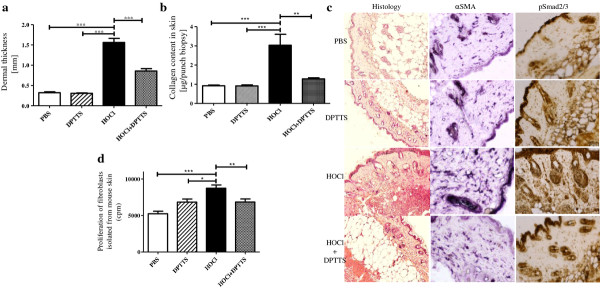
***In vivo *effects of DPTTS on skin fibrosis in HOCl mice (*n*** **= 8 mice per group). (a)** Dermal thickness as measured in the injected area. **(b)** Collagen content in the skin as measured by the Sircol method. **(c)** Representative tissue sections of dermal fibrosis (H&E staining), α-SMA, and pSmad 2/3 expressions (immunohistochemistry) in the injected areas. Magnification is × 50 (Olympus DP70 Controller). **(d)** Proliferation of fibroblasts from normal and HOCl mice treated or not with DPTTS. Cellular proliferation (cpm) was measured by thymidine incorporation. Values are expressed as mean ± SEM of data gained from all mice in the experimental or control groups. *P < 0.05; **P < 0.01; ***P < 0.001.

**Figure 4:**
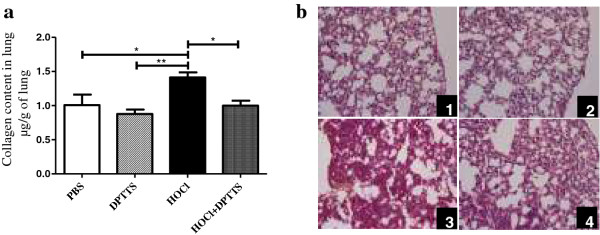
***In vivo *effect of DPTTS on lung fibrosis in HOCl mice (*n*** **= 8 mice per group). (a)** Collagen content in the lungs was measured by the Sircol method. **(b)** Representative lung sections from mice injected with (1) PBS, (2) DPTTS, (3) HOCl, and (4) HOCl + DPTTS. (H & E staining; magnification × 50; Olympus DP70 Controller). Values are expressed as mean ± SEM of data gained from all mice in the experimental or control groups. *P < 0.05; **P < 0.01.

HOCl-induced SSc is associated with an increase in dermal thickness that is significantly reduced by DPTTS (*P* < 0.001 versus untreated HOCl mice; Figure 3a). These results were confirmed by the histopathologic analysis of the skin of PBS and HOCl mice treated or not with DPTTS (Figure 3c). *In vivo,* DPTTS significantly reduced the accumulation of type I collagen induced by HOCl in the skin (*P* < 0.01; Figure 3b) and in the lung (*P* < 0.05; Figure 4a) versus untreated HOCl mice. Histopathologic analysis of lung biopsies stained with hematoxylin and eosin (Figure 4b) confirmed the reduction in lung fibrosis in HOCl mice treated with DPTTS. Moreover, the *ex vivo* proliferation rate of fibroblasts isolated from HOCl mice was significantly reduced by *in vivo* treatment with DPTTS (8,729 cpm ± 445 versus 6,842 cpm ± 420; *P* < 0.01; Figure 3d).

### DPTTS reduced the expression of αSMA and pSmad2/3 in HOCl mice

The expression of αSMA was significantly higher in the skin of HOCl mice than in PBS mice (22.6% ± 1.6% positive area in HOCl mice versu 10.01% ± 0.8%, in PBS mice; *P* < 0.01; Figure 3c). DPTTS decreased the expression of α-SMA by 40% in HOCl mice (13.7% ± 0.4% positive area versus 22.6% ± 1.6% positive area in untreated HOCl mice; *P* < 0.01; Figure 3c). The level of expression of pSmad 2/3, a key protein involved in TGF-β-induced fibrogenesis, was higher in HOCl mice than in PBS controls (24.5% ± 1.4% positive area in HOCl mice versus 5.8% ± 0.4% positive area in PBS mice; *P* < 0.01; Figure 3c). *In vivo* administration of DPTTS reduced pSmad2/3 expression in HOCl mice (7.2% ± 2.1% positive area versus 24.5% ± 1.4% positive area in untreated HOCl mice; *P* < 0.01; Figure 3c).

### DPTTS decreased the serum concentration of AOPP and anti-DNA topoisomerase-1 Abs in SSc mice

**Figure 5:**
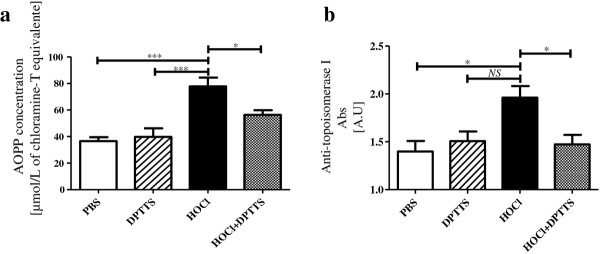
***In vivo*, DPTTS inhibits the production of autoantibodies and exerts beneficial effects on local and systemic oxidative stresses in HOCl mice. (a)** Serum AOPP levels (micromolar chloramine T equivalents). **(b)** Levels of anti-DNA topoisomerase-1 Abs measured with ELISA. Values are expressed as mean ± SEM of data gained from all mice in the experimental and control groups. *P < 0.05; ***P < 0.001.

Advanced oxidation protein products (AOPPs), a marker of systemic oxidative stress, were increased in the sera of HOCl mice compared with PBS mice (*P* < 0.001, Figure 5a). DPTTS reduced the levels of AOPP by 28% in HOCl mice versus untreated HOCl mice (*P* < 0.05; Figure 5a). The sera of HOCl mice contained significantly higher levels of anti-DNA-topoisomerase-1 abs than did the sera from PBS mice (1.96 A.U. ± 0.1 versus 1.39 A.U. ± 0.11; *P* < 0.05; Figure 5b). DNA-topoisomerase-1 abs were significantly decreased in the sera from HOCl mice treated with DPTTS compared with untreated HOCl mice (1.47 A.U. ± 0.09 versus 1.96 A.U. ± 0.1; *P* < 0.05; Figure 5b).

### DPTTS decreased the counts of B cells and the proliferation rate of B and T cells in HOCl mice

**Figure 6:**
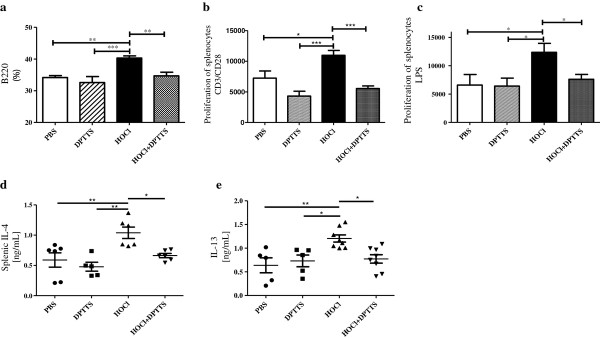
**Immunomodulating properties of DPTTS in mice with HOCl-induced SSc. (a)** Splenic B-cell numbers (B220 epitope), as assessed with flow cytometry. **(b)** Proliferation of splenic T cells activated by anti-CD3/CD28. **(c)** Proliferation of splenic B cells activated by LPS. Results are expressed in cpm. **(d, e)** Concentrations of IL-4 and IL-13 in supernatants of ConA-activated splenic T cells. Values are expressed as mean ± SEM of data gained from all mice in the experimental and control groups. *P < 0.05; **P < 0.01; ***P < 0.001.

We next tested the effects of DPTTS on spleen cell populations. Intradermal injection of HOCl significantly increased the number of splenic B cells in SSC mice compared with normal mice (40.3% ± 0.6% versus 34.1% ± 0.61%; *P* < 0.01; Figure 6a). DPTTS decreased the number of splenic B cells by 16% in HOCl mice compared with untreated HOCl mice (34.7% ± 1.1% versus 40.3% ± 0.6%; *P* < 0.01; Figure 6a).

We also investigated the proliferation rate of splenic T cells after stimulation with precoated anti-CD3/CD28 mAb, and of B cells after stimulation with LPS. T and B cells isolated from HOCl mice had higher proliferation rates than did T and B cells isolated from normal mice. T cells isolated from HOCl mice treated with DPTTS and stimulated *ex vivo* by an anti-CD3 mAb displayed a lower proliferation rate than did T cells obtained from untreated HOCl mice and stimulated under the same conditions (5,538 cpm ± 427 versus 10,967 cpm ± 786; *P* < 0.001; Figure 6b). B cells isolated from HOCl mice treated with DPTTS and stimulated with LPS also displayed a lower proliferation rate than did B cells obtained from untreated HOCl mice (7,625 cpm ± 851 versus 12,380 cpm ± 1,572; *P* < 0.05; Figure 6c).

### *In vivo* administration of DPTTS reduced the production of IL-4 and IL-13 in HOCl mice

HOCl mice had a higher serum concentration of IL-4 and IL-13 than did PBS-treated mice (1.03 ± 0.09 ng/ml versus 0.58 ± 0.1 ng/ml; *P* < 0.01 for IL-4, Figure 6d; 1.2 ± 0.07 ng/ml versus 0.63 ± 0.15 ng/ml; *P* < 0.01 for IL-13; Figure 6e). DPTTS decreased the levels of IL-4 in HOCl mice by 37% (*P* < 0.05; Figure 6d), and of IL-13 by 36% (*P* < 0.05; Figure 6e).

## Discussion

In the present study, we showed that the natural organosulfur compound, DPTTS, prevents the development of fibrosis in a murine model of chemically induced systemic sclerosis.

DPTTS is able to increase the intracellular level of ROS to generate a lethal oxidative burst in fibroblasts from mice with HOCl-induced SSc. The cytotoxic effect of DPTTS is observed only in diseased fibroblasts, not in healthy fibroblasts that display a normal level of endogenous reduced GSH and low levels of H_2_O_2_. Our results are in agreement with previous studies on polysulfides showing a prooxidant effect of these molecules. Indeed, in cancer cells that constitutively produce high amounts of ROS, diallyl-polysulfides further increase ROS generation, causing ß-tubulin oxidation, disruption of the microtubule network, and finally apoptosis [15,16].

Similarly, we showed that the organotelluride catalyst (PHTE)_2_NQ and arsenic trioxide molecules that increase the levels of ROS in activated fibroblasts of HOCl mice ameliorate the fibrosis in these animals through mechanism similar to that of DPTTS [6,15]. The protective effects of NAC, a GSH precursor, that neutralizes the cytotoxicity of DPTTS in HOCl fibroblasts, and the opposite effect of BSO, which depletes GSH, emphasize the role of the GSH pathway in the cytotoxicity of DPTTS.

A paradoxic effect of the prooxidative molecule DPTTS is the decrease in the serum concentration of AOPP observed in HOCl mice. This can be explained by the selective destruction of diseased fibroblasts, which chronically produce high levels of ROS that oxidize proteins of the skin, in particular, DNA topoisomerase-1 [7]. Because oxidized DNA topoisomerase-1 is one of the autoantigens responsible for the breach of tolerance in SSc, DPTTS indirectly abrogates the autoimmune reaction through the selective and early destruction of diseased fibroblasts.

DPTTS also downregulates the phosphorylation of Smad2/3 and contributes to decreasing the accumulation of type I collagen in the skin of mice with HOCl-induced SSc. Smad2 and Smad3 are transcription factors that are overexpressed in human SSc fibroblasts, as well as in fibroblasts from HOCl mice. Phosphorylated Smad2/3 activates genes coding for type I collagen, which leads to fibrosis in several organs [15,16]. In addition, TGF-β, which induces Smad2/3 phosphorylation, is inhibited by a thiol antioxidant-NAC, GSH, and L-cysteine, thus highlighting the role of H_2_O_2_ in the activation of the Smad2/3 pathway [17]. Therefore, in HOCl-induced SSc, the selective depletion of fibroblasts overproducing ROS by DPTTS decreases the number of cells with high levels of phosphorylated Smad2/3.

Other features of SSc in patients are an abnormal activation of immune T and B cells, the presence of inflammatory infiltrates (especially of CD4^+^ T cells) in the skin and in the lungs, along with increased levels of various proinflammatory and profibrotic cytokines [5,18]. DPTTS exerts an immunoregulatory effect in HOCl mice by limiting the expansion of B cells, and reducing the hyperproliferation of CD3/CD28-activated T cells and the proliferation of LPS-activated B cells. The biologic effect of garlic-derived organosulfur compounds on leukocytes has been a matter of controversy. Some reports describe immunostimulatory properties [19], whereas others highlight cytotoxic effects on lymphocytes [20] through their prooxidative activity [19,21]. In our hands, the immunomodulating properties could be related to the addition of the ROS overproduced in autoreactive B and T cells and of the ROS induced by DPTTS, as previously in HOCl mice treated with (PHTE)_2_NQ or arsenic trioxide [6,15,22]. The immunomodulatory properties of DPTTS are also characterized by a decrease in the splenic production of IL-4 and IL-13 in HOCl mice treated with this molecule. This effect on profibrotic cytokines, elevated in the skin and in the serum of patients with SSc [18,23], can explain, at least in part, the antifibrotic effects of DPTTS observed in HOCl mice.

## Conclusions

DPTTS, an organosulfur compound ubiquitous in plants of the genus *Allium*, prevents skin and lung fibrosis in the mouse through the selective killing of diseased fibroblasts. With a mouse model of systemic sclerosis, *in vivo* treatment with DPTTS indicates a potential new strategy of treatment of systemic sclerosis. More extensive *in vitro* and *in vivo* studies are now required in human tissues to evaluate its preclinical applications in connective tissue disorders.

## Abbreviations

AOPP: Advanced oxidation protein product; BSO: Buthionine sulfoximine; DAB: Diaminobenzidine tetrahydrochloride; DPTTS: Dipropyltetrasulfide; FACS: Fluorescence-activated cell sorting; GSH: Glutathione; IL: Interleukin; LPS: Lipopolysaccharide; NAC: *N*-acetylcysteine; OD: Optical density; PI: Propidium iodide; ROS: Reactive oxygen species; SSc: Systemic sclerosis; α-SMA: α-smooth muscle actin.

## Competing interests

Dipropyltetrasulfide (DPTTS) was synthesized and provided by Awais Anwar from ECOSpray in the UK.

## Authors’ contributions

All authors were involved in drafting the article or revising it critically for important intellectual content, and all authors approved the final version to be published. Dr. FB had full access to all of the data in the study and takes responsibility for the integrity of the data and the accuracy of the data analysis. Study conception and design were provided by WM, VJ, NK, AS, PGW, PE, CJ, BW, and FB; acquisition of data by WM, VJ, CN, NK, AS, CC, AA, PGW, PE, BW, and FB; and analysis and interpretation of data by WM, VJ, PGW, PE, CJ, BW, and FB.
